# When the Learner Is the Expert: A Simulation-Based Curriculum for Emergency Medicine Faculty

**DOI:** 10.5811/westjem.2019.11.45513

**Published:** 2019-12-19

**Authors:** Emily S. Binstadt, Rachel A. Dahms, Amanda J. Carlson, Cullen B. Hegarty, Jessie G. Nelson

**Affiliations:** * University of Minnesota, Regions Hospital Emergency Department, St. Paul, Minnesota; † St. Mary’s Medical Center Essentia Health, Department of Emergency Medicine, Duluth, Minnesota

## Abstract

Emergency physicians supervise residents performing rare clinical procedures, but they infrequently perform those procedures independently. Simulation offers a forum to practice procedural skills, but simulation labs often target resident learners, and barriers exist to faculty as learners in simulation-based training. Simulation-based curricula focused on improving emergency medicine (EM) faculty’s rare procedure skills were not discovered on review of published literature. Our objective was to create a sustainable, simulation-based faculty education curriculum for rare procedural skills in EM. Between 2012 and 2019, most EM teaching faculty at a single, urban, Level 1 trauma center completed an annual two-hour simulation-based rare procedure lab with small-group learning and guided hands-on instruction, covering 30 different procedural education sessions for faculty learners. A questionnaire administered before and after each session assessed EM faculty physicians’ self-perceived ability to perform these rare procedures. Participants’ self-reported confidence in their performance improved for all procedures, regardless of prior procedural experience. Faculty participation was initially mandatory, but is now voluntary. Diverse strategies were used to address barriers in this learner group including eliciting learner feedback, offering continuing medical education credits, gradual roll-out of checklist assessments, and welcoming expertise of faculty leaders from EM and other specialties and professions. Participants perceived training to be most helpful for the most rarely-encountered clinical procedures. Similar curricula could be implemented with minimal risk at other institutions.

## BACKGROUND

Academic emergency physicians are expected to perform rarely-occurring emergency clinical procedures proficiently and to teach these procedures to residents. Faculty in emergency medicine (EM) training programs may perform procedures less frequently than other emergency physicians, as they prioritize learner hands-on procedural exposure over their own opportunity to practice. Infrequent and unpredictable procedures are difficult to study.

Simulation allows learners to train for high-risk, low-frequency clinical events on a predictable timetable.^1^ Although many EM residency programs use simulation-based learning for procedural training,^2^ simulation has been infrequently used for faculty learners. This may be due to lack of protected time in faculty schedules, a potentially judgmental environment surrounding procedural competence, lack of faculty comfort with simulation-based learning, and fear of exposing incompetence to peers.^3^ Faculty often obtain continuing medical education (CME) training from passive learning or large-group settings, which changes performance less than hands-on learning.^4^–^6^

## OBJECTIVES

This work describes the development of a novel curriculum for EM faculty in a small group, hands-on, non-threatening, simulation-based learning environment to improve self-rated confidence with rare EM procedures. This course was refined over eight years, such that a novel procedural curriculum for academic EM faculty has emerged.

## CURRICULAR DESIGN

### Probblem Identification and General Needs Assessment

Some EM procedures are time-dependent and potentially life-saving. Increased practice and improved confidence performing these procedures may increase the likelihood that faculty will attempt and perform these procedures well. Research using faculty physicians as the study subjects in any procedural skills labs is quite limited.^7^–^14^ This curriculum is novel in that it focused on EM faculty as learners, and it focused on rare procedures.

### Targeted Needs Assessment

Faculty discussions in staff meetings and multiple ad hoc discussions revealed a list of EM procedures faculty members would be interested in practicing. Initial procedures in 2012 were lateral canthotomy, ultrasound-guided, internal jugular central venous access, resuscitative thoracotomy, and rescue airway techniques.

### Goals and Measureable Objectives

Recognizing the difficulty in assessing clinical outcomes for procedures performed infrequently, this project’s main objective was to improve the self-rated confidence levels of EM faculty members for performing rare procedures.

### Educational Strategies

Simulation centers are disproportionately used by trainees, likely due in part to challenges with faculty engagement, simulation center funding,^15^ generational gap in comfort and experience with simulation technology, anxiety about performing procedures in front of colleagues, and reluctance to donate time to participate in additional training or assessment sessions.^16^ This project circumvented some of these barriers by purposefully avoiding high-stakes assessment and focusing exclusively on a low-stakes, non-threatening, training environment. More objective assessments, such as checklists, were added after several years, once faculty buy-in and psychological safety regarding the activity had been established.

### Implementation

The intervention was a two-hour, simulation-based training session, repeated two to three times per year to allow all faculty to attend one session. The department head initially mandated attendance but not survey data collection. Each session included four procedural stations through which groups of two to four learners rotated. Each station focused on a different procedure. Initial procedures were chosen by consensus among the authors and simulation staff, favoring high-yield procedures with availability of reasonable simulation models and instructors. Learners had no advance notice of the procedures to preclude preparation for a specific procedure. Learners obtained CME credit for participating. Instructors were faculty volunteers. Each session was heavily focused on hands-on practice for participants, with brief discussion of procedural steps, indications and contraindications, and common pitfalls. Formative feedback and peer discussion were encouraged.

**Population Health Research Capsule**

What do we already know about this issue?

*Emergency medicine (EM) faculty may benefit from hands-on simulation-based procedural training, but lack regular opportunities to perform and practice EM procedures*.

What was the research question?

Does small-group, hands-on, simulation-based training improve EM faculty participants’ procedural performance confidence?

What was the major finding of the study?

*Participants improved self-assessed confidence in performing 30 different emergency procedures over 8 years*.

How does this improve population health?

*This procedural curriculum for EM faculty appears sustainable and effective in promoting procedural practice with guided feedback, and is low-risk and accessible*.

## IMPACT/EFFECTIVENESS

### Evaluation and Feedback

Self-rated confidence in procedural skill for selected procedures was rated on a visual analog scale (VAS; 100 millimeters) pre- and post-session (Appendix A). Anonymous written survey responses about impressions after the session were nearly universally positive. Response rate for surveys was 95%. Faculty’s self-reported confidence to perform each procedure improved for all 30 procedures (Appendix B). Faculty with higher pre-simulation experience with a procedure still demonstrated significant improvement in confidence scores.

At faculty and department head request, this training has been repeated annually for eight years, with evolution in the procedures taught. This curriculum has covered 30 different emergency procedures. Topics are chosen annually based on faculty requests, recent quality improvement initiatives, changing equipment and technology. This has proved to be a valuable venue for faculty education in general, with continued attendance even when no longer mandated and anticipated expansion to include more community-based faculty learners. Particularly time-critical procedures such as resuscitative thoracotomy, lateral canthotomy, and perimortem cesarean are repeated every few years. Summary data for these procedures is presented in Table 1. Additional recurrent themes in the curriculum are procedures related to airway technology and equipment, and methods for various types of intravenous access. Appendix B lists the 30 prior procedure training modules used in the curriculum. This program can be replicated at other institutions with EM faculty and commonly available simulation technology.

## LIMITATIONS

As with similar projects, there are limitations and lessons learned from this project. This effort’s impact is limited based on performance in a single center, with limited numbers of participants. Prioritizing feasibility and faculty acceptance, knowledge changes, timing of retention in confidence gains, and impact on clinical care were not studied here.

## CONCLUSION

These procedure labs will continue to be offered annually given positive faculty responses and continued interest. Anonymous satisfaction surveys for the curriculum demonstrate mainly “excellent” ratings of how it enhanced knowledge and ability to apply new strategies to clinical practice. Future studies are needed to determine whether participants with higher scores would actually perform better clinically, but exposing faculty to rare procedural practice in a standardized, non-threatening manner appears to be successful in increasing their perceptions of self-efficacy regarding their clinical competence. The resounding appreciation of this training among participants at all levels of previous procedural experience indicates that there is a desire for hands-on training with rare procedures among practicing emergency physicians. The risks of implementing this type of curriculum are low, and it may be preferred over traditional lecture formats. This curriculum offers an opportunity for faculty to participate in high-yield, low-stakes, sustainable, simulation-based learning to help attain and maintain expertise with rare clinical procedures.

## Supplementary Information





## Figures and Tables

**Table 1:** Emergency faculty physicians’ change in self-rated confidence in performance of three rarely-occurring procedures after procedural training.

Procedure	Average pre-training confidence	Average post-training confidence	Difference in confidence	Median # prior experiences	Range of # of prior experiences
Resuscitative thoracotomy 2012	44mm	66mm	22mm	3	0–190
Resuscitative thoracotomy 2015	61mm	80mm	19mm	4	0–20
Resuscitative thoracotomy 2019	67mm	84mm	17mm	6	3–102
Cricothyroidotomy	72mm	87mm	15mm	6	0–58
Lateral canthotomy 2012	30mm	66mm	36mm	0	0–5
Lateral canthotomy 2017	65mm	83mm	18mm	3	0–14
Peri-mortem cesarean section 2013	31mm	72mm	41mm	0	0–5
Peri-mortem cesearean section 2017	34mm	74mm	40mm	4	0–14

Physicians scored their confidence levels pre- and post-training on a 100mm visual analog scale. Three of the four procedures presented here were repeated in successive years, as labeled. The median number of physician-estimated personal prior experiences listed includes animal lab, cadaver lab, simulation lab, and clinical patient experiences. Despite prior simulation experience with the procedure, confidence continued to improve after successive training sessions. Lateral canthotomy confidence appeared more sustained than did confidence for peri-mortem cesarean section. Despite higher confidence scores pre-training for cricothyroidotomy, post-training scores still increased.

*mm*, millimeter.
