# Supplementary material for: When the Learner Is the Expert: A Simulation-Based Curriculum for Emergency Medicine Faculty
Source: West J Emerg Med. 2019 Dec 19;21(1):141–4. doi: 10.5811/westjem.2019.11.45513 (PMC6948691; doi:10.5811/westjem.2019.11.45513)
Supplement: Supplementary file 1 [file wjem-21-141-s001.docx]

**Appendix A.** Example of a pre- and post-training survey.

**Pre lab Survey 2017 Participant #_____**

**For ALL questions below, please indicate (S) behind your # if done in a simulation lab, (C) in a cadaver lab, and (A) in an animal lab, and (H) if done on a live human**

**For Perimortem C-section:**

How many times have you primarily preformed the procedure? ___A ___S ___C ___H

Assisted? __A ___S ___ C___H

Observed? ___A ___S ___C ___H

Taught? ___A ___S ___C ___H

Please make a vertical mark on the line below, indicating how you currently feel about your ability to efficiently perform the procedure?

Not Confident Somewhat Confident Very Confident

Please make a vertical mark indicating how you currently feel about your ability to perform the procedure safely for the patient?

Not Confident Somewhat Confident Very Confident

Please make a vertical mark indicating how you currently feel about your ability to teach the procedure?

Not Confident Somewhat Confident Very Confident

**For Lateral Canthotomy:**

How many times have you primarily preformed the procedure? ___A ___S ___C ___H

Assisted? __A ___S ___ C___H

Observed? ___A ___S ___C ___H

Taught? ___A ___S ___C ___H

Please make a vertical mark on the line below, indicating how you currently feel about your ability to efficiently perform the procedure?

Not Confident Somewhat Confident Very Confident

Please make a vertical mark indicating how you currently feel about your ability to perform the procedure safely for the patient?

Not Confident Somewhat Confident Very Confident

Please make a vertical mark indicating how you currently feel about your ability to teach the procedure?

Not Confident Somewhat Confident Very Confident

**For Peritonsillar Abscess I&D:**

How many times have you primarily preformed the procedure? ___A ___S ___C ___H

Assisted? __A ___S ___ C___H

Observed? ___A ___S ___C ___H

Taught? ___A ___S ___C ___H

**1)** Please make a vertical mark on the line below, indicating how you currently feel about your ability to efficiently perform the procedure?

Not Confident Somewhat Confident Very Confident

**2)** Please make a vertical mark indicating how you currently feel about your ability to perform the procedure safely for the patient?

Not Confident Somewhat Confident Very Confident

**3)** Please make a vertical mark indicating how you currently feel about your ability to teach the procedure?

Not Confident Somewhat Confident Very Confident

**For Complete ED Eye Exams including slit lamp, corneal exam, retinal exam, and nerve exam:**

How many times have you primarily preformed the procedure? ___A ___S ___C ___H

Assisted? __A ___S ___ C___H

Observed? ___A ___S ___C ___H

Taught? ___A ___S ___C ___H

**1)** Please make a vertical mark on the line below, indicating how you currently feel about your ability to efficiently perform the procedure?

Not Confident Somewhat Confident Very Confident

**2)** Please make a vertical mark indicating how you currently feel about your ability to perform the procedure safely for the patient?

Not Confident Somewhat Confident Very Confident

**3)** Please make a vertical mark indicating how you currently feel about your ability to teach the procedure?

Not Confident Somewhat Confident Very Confident

**Post Lab Survey Participant # _______**

**For Perimortem C-section:**

Please make a vertical mark on the line below, indicating how you currently feel about your ability to efficiently and successfully perform the procedure?

Not Confident Somewhat Confident Very Confident

Please make a vertical mark indicating how you currently feel about your ability to perform the procedure safely for the patient?

Not Confident Somewhat Confident Very Confident

Please make a vertical mark indicating how you currently feel about your ability to teach the procedure?

Not Confident Somewhat Confident Very Confident

**For Lateral Canthotomy:**

Please make a vertical mark on the line below, indicating how you currently feel about your ability to efficiently perform the procedure?

Not Confident Somewhat Confident Very Confident

Please make a vertical mark indicating how you currently feel about your ability to perform the procedure safely for the patient?

Not Confident Somewhat Confident Very Confident

Please make a vertical mark indicating how you currently feel about your ability to teach the procedure?

Not Confident Somewhat Confident Very Confident

**For Peritonsillar Abscess I&D:**

Please make a vertical mark on the line below, indicating how you currently feel about your ability to efficiently perform the procedure?

Not Confident Somewhat Confident Very Confident

Please make a vertical mark indicating how you currently feel about your ability to perform the procedure safely for the patient?

Not Confident Somewhat Confident Very Confident

Please make a vertical mark indicating how you currently feel about your ability to teach the procedure?

Not Confident Somewhat Confident Very Confident

**For Complete ED Eye Exams including slit lamp, corneal exam, retinal exam, and nerve exam:**

Please make a vertical mark on the line below, indicating how you currently feel about your ability to efficiently perform the procedure?

Not Confident Somewhat Confident Very Confident

Please make a vertical mark indicating how you currently feel about your ability to perform the procedure safely for the patient?

Not Confident Somewhat Confident Very Confident

Please make a vertical mark indicating how you currently feel about your ability to teach the procedure?

Not Confident Somewhat Confident Very Confident
