# Supplementary material for: When the Learner Is the Expert: A Simulation-Based Curriculum for Emergency Medicine Faculty
Source: West J Emerg Med. 2019 Dec 19;21(1):141–4. doi: 10.5811/westjem.2019.11.45513 (PMC6948691; doi:10.5811/westjem.2019.11.45513)
Supplement: Supplementary file 2 [file wjem-21-141-s002.docx]

**Appendix B.** 30 Procedures taught in rare procedure lab for emergency medicine faculty, 2012-2019.

| Dental fracture repair  Extensor tendon repair  Fingertip rongeur and flap closure  Gastrostomy tube replacement HAZMAT/chemical decontamination  Hemodialysis or cooling catheter insertion  Intubation-Fiberoptic  Intubation-Retrograde  Lateral canthotomy  Neonatal intraosseous access  MN Tube  Neonatal Resuscitation | Pediatric failed airway/needle cricothyroidotomy/transtracheal jet ventilation  Pericardiocentesis  Perimortem cesarean  Peritonsillar abscess drainage Resuscitative thoracotomy  Slit lamp exam  Surgical cricothyroidotomy  Tracheostomy troubleshooting  Ultrasound-guided internal Jugular vein catheterization  Lumbar puncture  Tourniquet application  Vaginal delivery | Ultrasound-Rapid ultrasound in hypotension  Ultrasound-Right upper quadrant abdominal point-of-care  Ultrasound-Upper extremity regional nerve block  Ultrasound – Lower extremities regional nerve block  Umbilical vein catheterization  Chest Tubes (all types) |
| --- | --- | --- |
